# Effects of muscle relaxation on sustained contraction of ipsilateral remote muscle

**DOI:** 10.14814/phy2.12620

**Published:** 2015-11-26

**Authors:** Kouki Kato, Tasuku Watanabe, Kazuyuki Kanosue

**Affiliations:** 1 Faculty of Sport Sciences, Waseda UniversityTokorozawa, Japan; 2 Japan Society for the Promotion of ScienceChiyoda-ku, Japan

**Keywords:** Coordination, inhibition, multilimb movements

## Abstract

The objective of this study was to clarify the temporal change of muscle activity during relaxation of ipsilateral remote muscles. While participants maintained a constant right wrist extensor isometric force, they dorsiflexed the ipsilateral ankle from resting position or relaxed from dorsiflexed position in response to an audio signal. The wrist extensor force magnitude increased in the 0–400 msec period after the onset of foot contraction compared to that of the resting condition (*P *<* *0.05). On the other hand, wrist extensor force magnitude and electromyographic (EMG) activity decreased in the 0–400 msec period after the onset of ankle dorsiflexion compared to that of the resting condition (*P *<* *0.05). Our findings suggest that foot muscle relaxation induces temporal reduction in hand muscle EMG activity and force magnitude.

## Introduction

Complex multilimb movements such as playing the piano or drum require fine coordination, not only of muscle contraction but also of muscle relaxation. It is well known that muscle contraction influence movement/muscle contraction in another limb (remote effect). For example, when humans execute cyclic movements of ipsilateral upper and lower limbs, movement in one limb is affected by the movement of the other (Baldissera et al. 1982; Kelso and Jeka 1992; Carson et al. 1995). Moreover, simple muscle contraction induces enhancement of tendon reflex and increase in excitability in the primary motor cortex (M1) that controls remote muscles in the ipsilateral and contralateral limbs (Delwaide and Toulouse 1981; Miyahara et al. 1996; Zehr and Stein 1999; Tazoe et al. 2007). So far, studies on this remote effect focused only on muscle contraction, the remote effect of muscle relaxation has not been studied.

It has recently been demonstrated that an “active process” is involved in muscle relaxation (Toma et al. 1999, 2000; Motawar et al. 2012). That is, neuroimaging and neurophysiological studies utilizing transcranial magnetic stimulation (TMS), functional magnetic resonance imaging (fMRI), magnetoencephalography (MEG), and electroencephalography (EEG) have commonly suggested that the activation of M1 was involved in muscle relaxation as well as in muscle contraction (Rothwell et al. 1998; Toma et al. 1999, 2000; Alegre et al. 2003; Buccolieri et al. 2004; Motawar et al. 2012; Kato et al. 2015).

We have recently investigated the interaction between simultaneous contraction and relaxation in two different limbs. Relaxation of one limb induced a decrease in electromyogram (EMG) activity during simultaneous contraction in the other limb (e.g., hand relaxation decreased EMG activity during foot contraction), but the opposite was not observed; there was no effect of contraction in one limb on relaxation in a different limb (Kato et al. 2014). However, since we utilized phasic contraction in our previous study, the time course of this inhibitory effect of relaxation remains unclear. Thus, in the present study we examine the effect of foot contraction/relaxation on sustained ipsilateral hand muscle contraction. We hypothesized that relaxation of one foot muscle would induce temporal suppression of EMG activity of sustained contraction in the ipsilateral hand muscle in the period around relaxation onset.

## Materials and Methods

### Participants

Ten right-handed, healthy volunteers (eight men and two women, mean 21.9 ± 1.9 years old, range 20–25 years) without known neurological or psychiatric disease participated in the experiment. All participants gave their written informed consent. The experimental procedure was approved by the Human Research Ethics Committee of Waseda University and performed according to the Declaration of Helsinki.

### Recording

**Figure 1:**
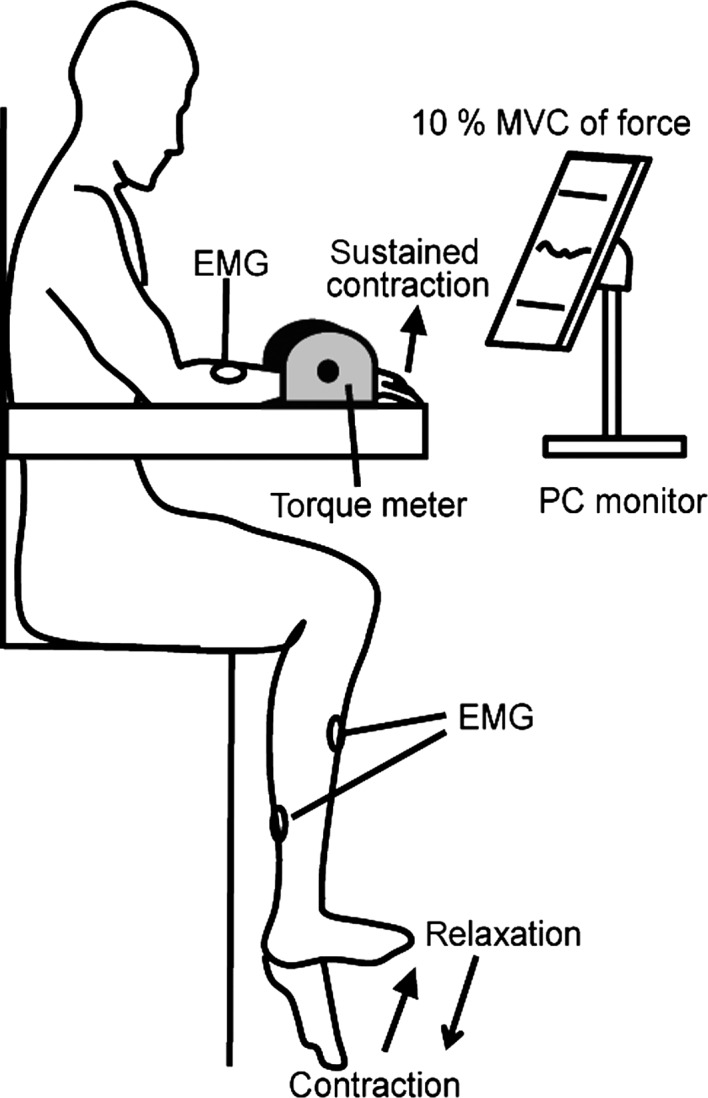
Illustration of the experimental setup.

**Figure 2:**
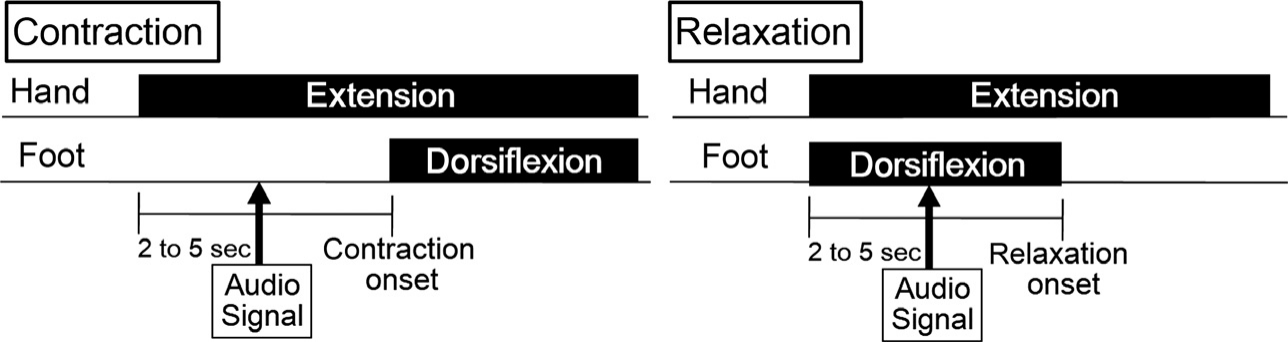
Schematic diagram of the contraction and relaxation tasks. Participants were asked to maintain a right-foot resting/dorsiflexed position, and to contract/relax as quickly as possible after an auditory stimulus.

A surface EMG was recorded from the right extensor carpi radialis (ECR), flexor carpi radialis (FCR), tibialis anterior (TA), and soleus (SOL) muscles, and disposable Ag–AgCl electrodes (1 cm diameter) were placed over the belly of the muscles at an interelectrode distance of 2 cm (Fig.1). Before the electrodes were attached, the area of skin was shaved and treated with alcohol to reduce interelectrode impedance. Interelectrode impedances and EMG signals for the four muscles were checked after placing the electrodes. Ankle joint angle was measured using goniometers (SG150, Biometrics, Newport, U.K.) that were secured at the lateral side of the ankle. The force magnitude of wrist extension was obtained by utilizing a torque meter (Fig.2; VTE-002R, VINE, Tokyo, Japan).

EMG signals were amplified (MEB-2216, Nihon Kohden, Tokyo, Japan) and filtered with a band-pass filter of 5–1500 Hz. Force magnitudes produced during wrist extension were amplified by a strain amplifier (DPM-611B, Kyowa, Tokyo, Japan). These data were recorded via an A/D converter system (Power lab 16/30, ADInstruments, Nagoya, Japan) at 4000 Hz for later analysis.

### Procedure

The participants sat with their right forearm supported in a horizontal position on an armrest (Fig.1). The experimenter confirmed that the right foot of the participant never touched the ground. The forearm was strapped to the armrest in a pronated position and attached to a torque meter. Participants controlled the isometric force magnitude of the wrist extensor at 10% of maximum voluntary contraction (MVC). At the beginning of the experiment, the participants were asked to perform wrist extensor MVC for 3 sec. Participants were verbally encouraged to achieve maximum force. MVCs were recorded three times. Each participant was then allowed more than 5 min of rest to avoid fatigue. The three MVCs were averaged, and 10% of the averaged MVC was calculated. The actual force magnitude exerted during each trial and the line representing the target force that the participant aimed to exert were displayed on a PC monitor which was positioned about 1 m away from each participant. The participants then practiced force control by matching the 10% MVC target force levels. There were two tasks: contraction and relaxation. In the contraction task, the participants were verbally informed to start and maintain the isometric extension force of their right hand at 10% MVC, and to completely relax their right foot in the resting position. Then, when a pure audio signal tone (SEIKO SQ100-77 Digital quartz metronome, signal duration; 40 msec) at a comfortable hearing level was presented through an earphone, the participants were instructed to dorsiflex their right foot to the maximum position as quickly as possible and not to change the hand extension. In the relaxation task, the participants were asked to start and maintain the right-hand extension force at 10% MVC, and to simultaneously maintain maximum right-foot dorsiflexion. Then, when an audio signal was presented, the participants were instructed to relax their right foot as quickly as possible. The maintenance period from the right-hand extension at 10% MVC to the audio signal was randomly set at 2–5 sec. Participants practiced contraction or relaxation of their right foot more than 10 times. If the experimenter confirmed unexpected EMG activity in the SOL during the relaxation task, the practice session was repeated until the EMG activity disappeared. Twenty trials of each task (contraction or relaxation), 40 trials in total, were randomly performed. To avoid fatigue, the intertrial interval was always longer than 10 sec. The participants took a 5-min break every five trials. The whole experiment lasted approximately 1 h.

### Data analysis

Dorsiflexor contraction and relaxation onsets in each trial were visually identified by an experimenter, based on the TA EMG (Buccolieri et al. 2004; Begum et al. 2005). For the contraction task, mean EMG activity of dorsiflexion was measured during the 600–800 msec period starting at contraction onset, assessed because stable EMG activities were obtained during the 600–800 msec period after changes in ankle angle terminated.

For the relaxation task, mean EMG activity of dorsiflexion was measured during the −200 to 0 msec period starting at the onset of the audio signal. The mean force magnitude of wrist extension was calculated in 200 msec bins (−400 to −200 msec, −200 to 0 msec, 0–200 msec, 200–400 msec, 400–600 msec, and 600–800 msec from contraction/relaxation onset) in each trial, then standardized by the values of −200 to 0 msec (baseline) from the audio signal onset. Likewise, root mean squares (RMSs) of ECR EMG activity were calculated for each of the above time bins; these RMS values were then standardized by the baseline value. Furthermore, the arithmetic mean of wrist extensor EMG activity was calculated. These values were averaged for all participants.

During the relaxation task, if a trial involved definite SOL activity greater than 100 *μ*V, the data from that trial were also excluded from analysis. The mean number of trials from which data were rejected was 3.1 ± 2.6 for the relaxation task.

For statistical analysis of the time course of EMG activity and force magnitude changes, the normality of the data distributions was initially assessed with the Shapiro–Wilks test. Since the distributions were found to be non-normal (*P* < 0.01), a nonparametric test was utilized to test for significance. ECR EMG activity and wrist extensor force magnitude for six bins during foot contraction/relaxation were compared to those in the baseline (baseline = 1) with the Wilcoxon signed-rank test (two-sided). These tests followed the protocol utilized in previous studies of muscle relaxation (Begum et al. 2005; Kato et al. 2015). Statistical significance was set at *P *<* *0.05.

## Results

The mean TA EMG activity during the contraction task (after dorsiflexion) and the relaxation task (before dorsiflexion) were 49.2 ± 7.1% MVC and 48.2 ± 6.9% MVC, respectively. The mean latencies of foot relaxation and contraction onset across all participants were 187 ± 18 msec and 193 ± 20 msec, respectively.

**Figure 3:**
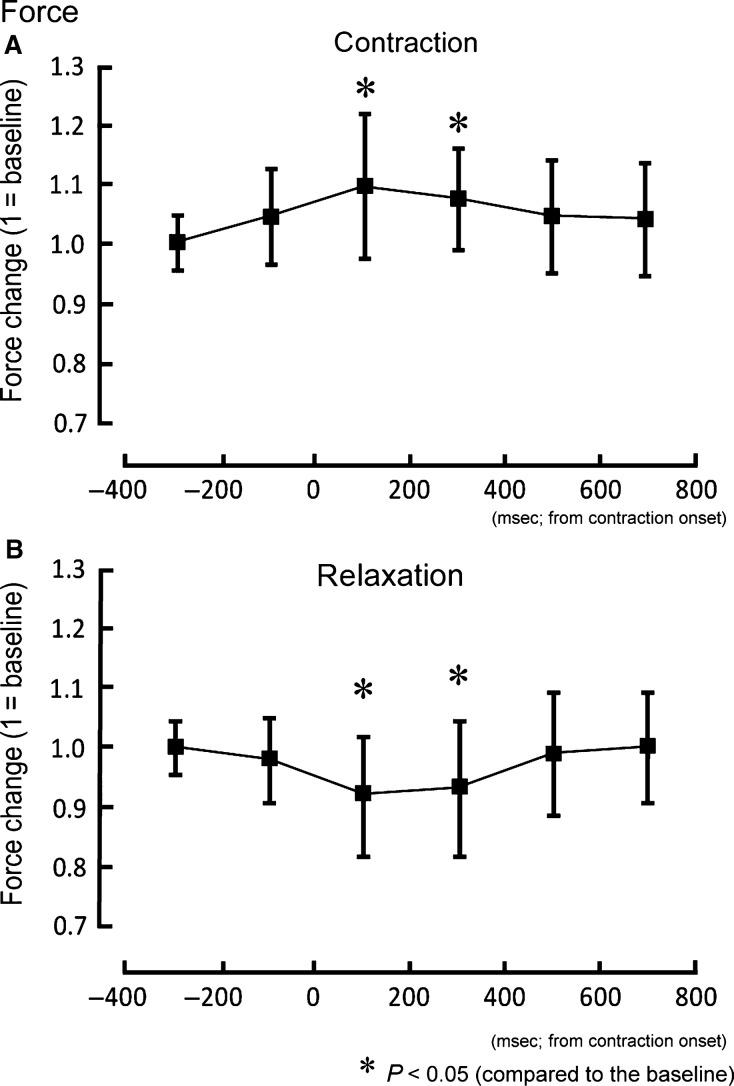
The mean force changes and standard deviation of the wrist extensor during contraction (A) and relaxation of foot (B). *P < 0.05 between the values and the baseline condition (1 = baseline).

**Figure 4:**
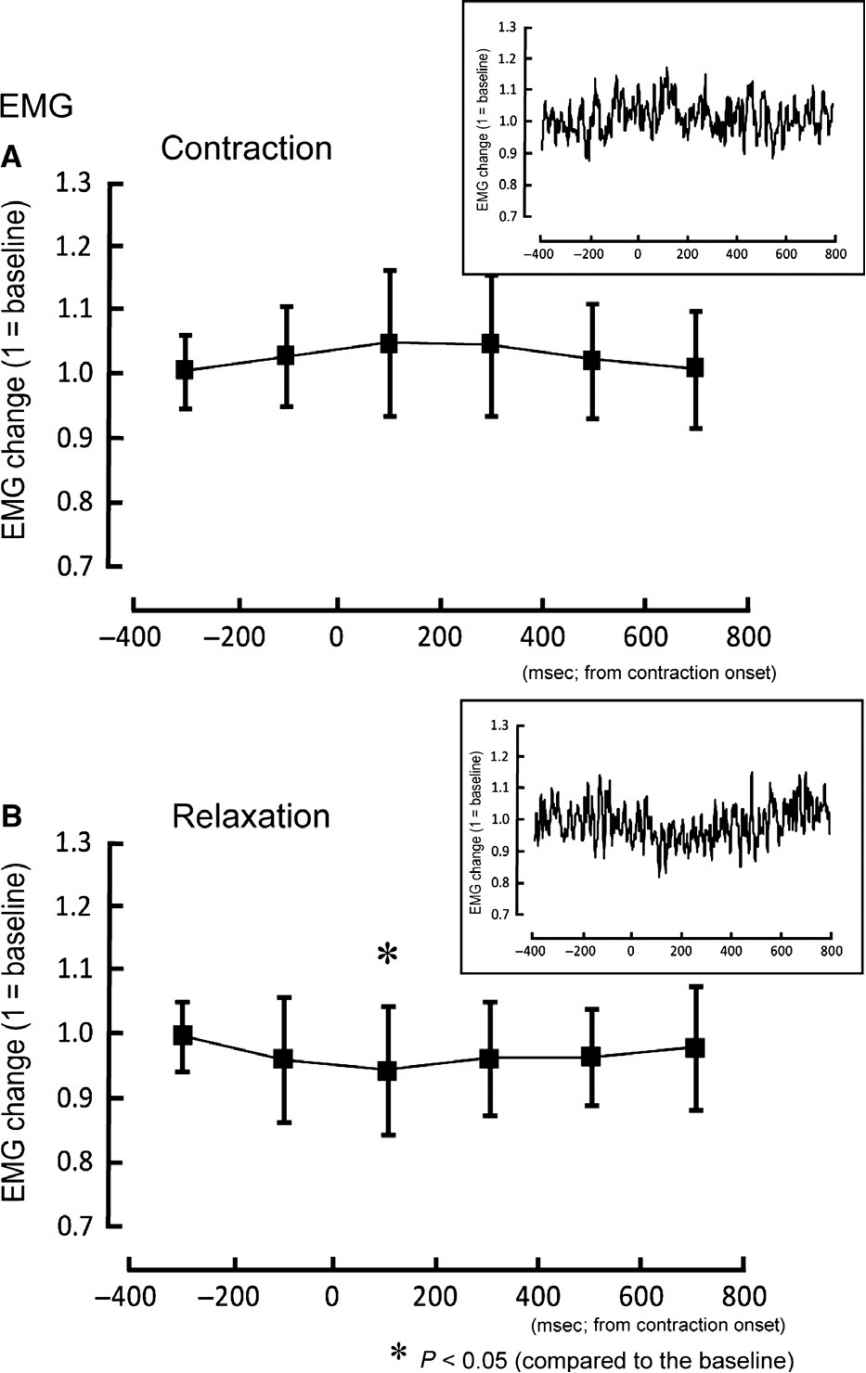
The mean EMG changes and standard deviation of the wrist extensor during contraction (A) and relaxation of foot (B). *P < 0.05 between the values and the baseline condition (1 = baseline). The insets show the arithmetic mean of wrist extensor EMG changes for all subjects.

The force magnitudes of hand contraction trials in the 0–200 msec (*Z* = 2.70, *P* < 0.05) and 200–400 msec (*Z* = 2.29, *P* < 0.05) intervals were significantly higher than the baseline (Fig.3A). On the other hand, in the relaxation trial hand force magnitudes in the 0–200 msec (*Z* = 2.19, *P* < 0.05) and 200–400 msec (*Z* = 2.80, *P* < 0.05) intervals were significantly lower than the baseline (Fig.3B). The ECR EMG activity in the 0–200 msec interval was significantly smaller (*Z* = 2.81, *P* < 0.05) than baseline (Fig.4B). For the contraction trials, no significant change was observed in EMG activity (Fig.4A).

Mean baseline background ECR EMG was 15.3 ± 3.5% MVC for contraction and 15.6 ± 3.5% MVC for relaxation.

## Discussion

The purpose of the present study was to investigate whether sustained isometric wrist extensor force would be affected by ipsilateral foot relaxation. When participants maintained a constant wrist extensor force, temporary changes in the force and ECR EMG activity were observed during both foot contraction and relaxation.

The force of wrist extension increased above baseline during ankle dorsiflexion (Fig.3). When a muscle is voluntarily contracted, corticospinal excitability of the target muscle increases (Ridding et al. 1995). Moreover, sustained ankle dorsiflexion induced an increase in corticospinal excitability of ipsilateral hand muscle (Tazoe et al. 2007), and the corticospinal hand extensor excitability temporarily increased with ipsilateral foot dorsiflexion (Borroni et al. 2004). The temporal increase in force magnitude in the present study was consistent with these previous observations, and could have been caused by the increase in corticospinal excitability of hand muscle.

Previous studies revealed that during muscle relaxation the corticospinal excitability of the muscle itself is decreased, not by the cessation of the muscle contraction excitatory process but by the involvement of an inhibitory mechanism within the M1 (Buccolieri et al. 2004; Motawar et al. 2012). Recently, Kato et al. (2014) conducted an experiment in which hand muscles contracted at the same time that the ipsilateral foot muscle, which had contracted up to that moment, relaxed. Under these conditions, EMG activity of hand contraction was reduced and the reaction time was longer than in the case when the hand muscle contracted alone (Kato et al. 2014). However, the time course of the inhibitory effect of muscle relaxation in one limb on muscle contraction of the other limb remains unclear. Moreover, since the simple reaction time of a muscle contraction in one limb is prolonged by simultaneous relaxation in the ipsilateral limb compared to the reaction time of a single limb contraction (Nissen and Bullemer 1987), the cognitive process required to accomplish the dual task (e.g., simultaneous hand relaxation and foot contraction) might have been involved in the remote effect. Although participants had to perform dual tasks in the present study also, one task was phasic and the other was tonic. Foot relaxation inhibited tonic hand contraction while foot contraction excited it, indicating that the suppressive effect of relaxation on remote muscle activity is not merely due to the influence of performing two tasks at the same time.

The same intracortical inhibitory mechanism within the M1 as was active during the decrease in corticospinal excitability of the relaxing muscle itself might be involved in the reductions in remote muscle EMG activity and force magnitude observed in the present study. Previous studies demonstrated that the GABA_A_ receptor mediated the intracortical inhibition of the target muscle which increased during relaxation (Ziemann et al. 1996; Buccolieri et al. 2004; Motawar et al. 2012). During foot relaxation in the present study, the inhibitory action within the M1 foot area might have spread to the hand area to cause the reduction of hand muscle EMG and force. However, since there is no anatomical connection between hand and foot areas in the M1 (Huntley and Jones 1991), some structures functionally above the M1 would have to be involved in the remote effect. Indeed, an fMRI study revealed that not only the M1, but also the supplementary motor area was activated during simple muscle relaxation as well as during muscle contraction (Toma et al. 1999). Although the present study does not answer the question of whether an inhibitory mechanism was involved, such an inhibition might be present not only for target muscle, but also for remote muscles.

It has also been reported that the TMS-induced cortical silent period in the hand extensor was shortened during tonic contraction of the foot dorsiflexor (Sohn et al. 2005; Tazoe et al. 2007), meaning that cortical excitability is increased as a result of suppressing the intracortical inhibitory process (i.e., disinhibition occurred) (Fuhr et al. 1991; Chen et al. 1999; Terao and Ugawa 2002). In the present study, therefore, disinhibition of the M1 area controlling the hand extensor might occur during static foot dorsiflexion before the foot began to relax. Under the condition in which disinhibition occurred, participants had to maintain the force level of hand extension. Thus, we speculate that disappearance of disinhibition at the termination of foot dorsiflexion (relaxation) induced the reduction of force magnitude.

The Go/No-go task has been widely utilized to investigate the inhibitory processes of motor control and the time course of process activity (Waldvogel et al. 2000; Nakata et al. 2006). In the Go/No-go task, participants respond to one cue (the Go stimulus), and they are required to not respond to another cue (the No-go stimulus). A previous study showed that during a No-go trial the corticospinal excitability of the target muscle decreased approximately 200 msec after the cue signal (Yamanaka et al. 2002). As for the remote muscle, corticospinal excitability of hand muscle in one limb which was not involved in the Go/No-go task was suppressed in the period approximately 200–400 msec after the cue signal for the No-go trial with the contralateral hand (Leocani et al. 2000). For ipsilateral hand and foot, moreover, the reduction in corticospinal excitability for resting foot muscle was observed with the peak latency at 400 msec after the cue for No-go trial with the hand (Badry et al. 2009). Thus, the decrease in corticospinal excitability of a remote muscle might be delayed by 0–200 msec compared to that of target muscle itself. As for muscle relaxation, the increase in intracortical inhibition occurred just before the target muscle relaxation onset (Buccolieri et al. 2004; Motawar et al. 2012). In the present study, the temporary reduction in force magnitude and EMG activity during the relaxation of a remote muscle was observed in the 0–200 msec period after relaxation onset (Figs.3, 4). From the fact that the latency of the remote effect of muscle relaxation is comparable to the latency of the remote effect of the No-go task, we speculate that a similar mechanism might be involved in these two tasks of muscle relaxation and inactivation.

It was likely that “attention” was involved in the reduction in force during relaxation. Since the relaxation was an unfamiliar motion, the participants might have paid more attention to relaxation than to contraction. As far as attention was concerned, contraction in one limb and relaxation in the other limb might be a dual task. Kahneman (1973) proposed that since we have limited signal processing capacity in the brain, we have to divide our attention by differentially allocating the amount of attention we pay to each simultaneous multiple task. Therefore, it was likely that the participants divided the amount of attention paid to sustained contraction of their hand with attention paid to “unfamiliar” relaxation. This would have suppressed the sustained contraction level of the other limb. Further experimentation is needed to elucidate the involvement of attention in the remote effects of muscle relaxation.

## Conclusion

The wrist extensor force magnitude increased after the onset of foot contraction compared to that of the resting condition. On the other hand, wrist extensor force magnitude and EMG activity decreased after the onset of ankle dorsiflexion compared to that of the resting condition. This finding suggests that foot muscle relaxation induces temporal reduction in hand force magnitude and muscle activity.
